# Development and evaluation of a leadership training program for public health emergency response: results from a Chinese study

**DOI:** 10.1186/1471-2458-8-377

**Published:** 2008-10-30

**Authors:** Chongjian Wang, Sheng Wei, Hao Xiang, Jing Wu, Yihua Xu, Li Liu, Shaofa Nie

**Affiliations:** 1 Department of Epidemiology and Biostatistics, School of Public Health, Tongji Medical College, Huazhong University of Science and Technology, Wuhan, PR China; 2 Department of Epidemiology and Biostatistics, College of Public Health, Zhengzhou University, Zhengzhou, Henan, PR China

## Abstract

**Background:**

Since the 9/11 attack and severe acute respiratory syndrome (SARS), the development of qualified and able public health leaders has become a new urgency in building the infrastructure needed to address public health emergencies. Although previous studies have reported that the training of individual leaders is an important approach, the systemic and scientific training model need further improvement and development. The purpose of this study was to develop, deliver, and evaluate a participatory leadership training program for emergency response.

**Methods:**

Forty-one public health leaders (*N *= 41) from five provinces completed the entire emergency preparedness training program in China. The program was evaluated by anonymous questionnaires and semi-structured interviews held prior to training, immediately post-training and 12-month after training (Follow-up).

**Results:**

The emergency preparedness training resulted in positive shifts in knowledge, self-assessment of skills for public health leaders. More than ninety-five percent of participants reported that the training model was scientific and feasible. Moreover, the response of participants in the program to the avian influenza outbreak, as well as the planned evaluations for this leadership training program, further demonstrated both the successful approaches and methods and the positive impact of this integrated leadership training initiative.

**Conclusion:**

The emergency preparedness training program met its aims and objectives satisfactorily, and improved the emergency capability of public health leaders. This suggests that the leadership training model was effective and feasible in improving the emergency preparedness capability.

## Background

Since the 9/11 attack, mad cow disease, severe acute respiratory syndrome (SARS), and avian influenza outbreaks, public health emergencies have become an utmost threat to communities worldwide. Moreover, because public health provides population-focused services to entire communities rather than individualized care [1,2], an increasing need exists for public health personnel capable of leading efforts to ensure the effectiveness and quality of these services [3]. During the past decade, there has been a growing interest in improving the emergency preparedness capability of public health leaders in the United States and other countries [4-10]. Although the Chinese government carried out a series of emergency preparedness education and training programs to improve the capability of public health leaders after the SARS outbreak, it remains unclear if these training programs are feasible and effective in improving the capability of emergency preparedness.

Previous studies showed that the emergency preparedness capability of public health leaders was insufficient in China [11,12]. In order to change the current situation and to improve emergency preparedness capability of public health leaders, one emergency preparedness training program for public health leaders was developed and supported by the Ministry of Health (MOH) of the People's Republic of China and the World Health Organization (WHO). The study was carried out by Tongji Medical College Emergency Institute (TMCEI) from 2006 to 2007. The training was completed in 2006, and the follow-up survey was conducted 12 months later. The purpose of this article was to evaluate the effectiveness of a leadership training program in improving the emergency capability, and then to develop a participatory leadership training model for public health emergency response.

### Program development

**Figure 1:**
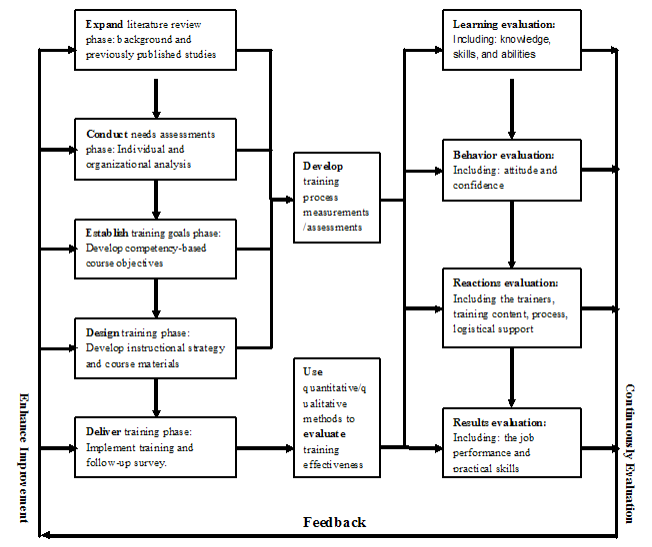
The public health leaders training model.

The program was adapted from a generic training system model [13]. The leadership training model emphasizes the major components of instructional design, including assessing, designing, delivering, and evaluating training (Fig. 1). The model is an integrated system with results from one phase influencing the next, so that a series of steps are followed when developing, implementing, and evaluating emergency preparedness training. This process begins with literature review and needs assessment, which enables the development of instructional objectives specifying what is to be achieved in the training, which, in turn, provides input for designing, delivering, and evaluating the effectiveness of the training program [13-15]. As Figure 1 shows, the model is a closed-loop system. Information resulting from evaluation of training effectiveness is used to determine whether the training met its previously defined aims and objectives. This information provides feedback necessary to modify future training system features by reassessing training needs, revising course objectives, or altering the delivery methods. The leadership training model is continually evolving, with results from previous programs being used to continuously improve future training programs [14].

## Methods and Subjects

### Aims and objectives

The aims and objectives of leadership training were carefully designed in consultation with the educational and training experts who have profound knowledge of the public health emergency response plan and the training program. The overall goals of the project were to: (1) develop and deliver a participatory leadership training program on responding to public health emergencies, and (2) test whether this pilot leadership training program prepares public health leaders to better respond to emergencies. The objectives of the training were subjected to continuous monitoring and evaluation during the training period.

### Participants and trainers

The research was approved by Tongji Medical College Ethics Committee, and all participants in the study agreed with the relevant training data for the study. Forty-three public health leaders from Health department or Centers for Disease Control and Prevention (CDC) in Hubei, Henan, Hunan, Jiangxi, and Anhui, participated in the leadership training program in 2006. There were 20 participants in the class held on July, 23 and in the class held in August. Each class lasted 14 days (112 hours). Two participants did not complete their training for reasons unrelated to the training, and they were not included in the evaluation (*N *= 41, 95.35% response rate). Trainers came from MOH, WHO, Chinese CDC, Fudan University, Wuhan University, and Huazhong University of Science and Technology. The selection of trainers was based on their expertise in the field of public health emergency response, related training programs and their involvement in continuous consultations on health service programs, both educational and promotional.

### Course and content

The training contents were designed with the American CDC's emergency preparedness core competencies for public health leaders/administrators as a framework [16-19]. Meanwhile, the investigators' and the course instructors' experiences and education in training programs were referenced. In brief, the training covered the following topics: (1) the definition of public health emergency; (2) the responsibilities of local, provincial, and governmental agencies during emergencies; (3) the role of public health leader during emergencies, (4) the public health information, roles, capacities, and legal authority to all emergency response partners; (5) reputation and relationship (trust) building, integrity, and credibility; (6) the ability to communicate effectively during emergencies; (7) the emergency response chain of command; (8) the emergency response protocols and management procedures, including the management of necessary supplies and equipment. These topics met the learning objectives displayed in table 1. The training contents were subjected to continuous monitoring and evaluation during the entire training period.

**Table 1:** Learning objectives: emergency preparedness training program for public health leader

**No**	**Core competencies for public health leader**
**1.**	**Describe **the public heath role in emergency response in a range of emergencies that might arise (*e.g., This department provides surveillance, investigation, and public information in disease outbreaks and collaborates with other agencies in biological, environmental in weather emergencies*).
2	**Recognize **unusual events that might indicate an emergency and **describe **appropriate action (*e.g., communicate clearly within the chain of command*.)
3	**Identify **limits to own knowledge/skill/authority and **identify **key system resources for referring matters that exceed these limits.
4	**Describe **his/her functional role(s) in emergency response and **demonstrate **his/her role(s) in regular drills.
5	**Identify **and **locate **the agency emergency response plan (*or the pertinent portion of the plan*).
6	**Explain **the interaction of central and local agencies and **describe **communication role(s) in emergency response (*media, within agency, general public and personal*)
7	**Evaluate **every emergency response drill/emergency response to identify needed internal/external improvements.
8	**Describe **the chain of command and management system (*"incident command system" or "similar protocol"*) for emergency response in the jurisdiction.
9	**Apply **creative problem solving and flexible thinking to unusual challenges within his/her functional responsibilities and **evaluate **effectiveness of all actions taken

### Format and process

Various training methods were used, including case studies, workshops, tutorials, seminars, group discussions, role-playing, drilling, and fieldwork. Formal lecturing was the method least used. The training center was equipped with modern audiovisual aids designed for training purpose. The training logistics and general services, such as transportation and housing, were provided free of charge to the participants.

### Evaluation design

The purpose of evaluating any program is to identify its strengths and weaknesses so that modifications can be made. This was especially true for this program which was designed to be innovative, relevant, flexible and not just a "one size fits all". The program was evaluated by two different methods: anonymous questionnaires and semi-structured interviews.

Through an anonymous questionnaire, the following outcomes were measured and investigated: individual basic information, knowledge levels, and self-assessment of skills regarding emergency preparedness. The questionnaire was designed by experts in the field of training programs and continuous consultation on emergency management. In order to assess the questionnaire, a pilot test was carried out among other public health leaders who did not participate in the training, and based on their feedback, subsequent modifications were made. A total of forty questions assessed participant's knowledge on the public health emergency competencies according to the "Core Public Health Worker Competencies for Emergency Preparedness and Response" [16] and "Ten Essential Public Health Services" [20], which consisted of basic public health science and culture knowledge, analytic/assessment knowledge, program planning and management knowledge. For a correct answer to a question, the participant received one point, whereas an incorrect answer received no points. Additionally, respondents were asked fifteen self-assessment questions so that the frequency of their use of each of the competencies could be measured [3]. Responses were rated on an ordinal scale (1 = "very low", 2 = "low", 3 = "Average", 4 = "high", 5 = "very high"). Participants completed the first measurement (pre-test, baseline) on the first day of training. The post-training measurement (post-test) was conducted at the end of the last day of training. For the third measurement (Follow-up test), the participants were mailed a copy of the survey with a self-addressed return envelope 12-months after completion of the program.

The training program was also subjected to continuous monitoring and evaluation through semi-structured interviews. The inclusion of the trainees in the evaluation process was extremely helpful in updating and modifying the program, for both the betterment of the program and the satisfaction of all the participants. The items addressed in the semi-structured interviews were as follows: (1) the scientific methods offered, (2) the technical material presented, (3) the performance of the trainers, (4) the benefits derived by the participant, (5) the use of the audiovisual aids, (6) the strengths and weaknesses of the session, (7) final critical comments and remarks, and (8) the training model. The forms were distributed at the end of each session to be completed anonymously by each participant. The forms were immediately analyzed and the results shown to the trainer who had conducted the session. If any deficiencies were revealed, the necessary modifications were made immediately. Evaluation of workshops and fieldwork was carried out in a similar fashion. Feedback of the results of the evaluation was given to the participants.

### Data analysis

The responses to the forty questions regarding emergency knowledge were reported as scores. The scores of self-assessment were derived for each domain by participants' response to all the frequency questions separately. Repeated-measures analysis of variance was used to test differences between pre-test, post-test, and follow-up test. The data from the semi-structured interviews was categorized and analyzed by three authors independently using the triangulation method, and their individual results were compared and discussed, and a consensus was reached. Data was analyzed by one way ANOVA with SPSS12.0 (SPSS. Inc., USA) for Windows. *P*-value of < 0.05 was used as the significance level.

## Results

### Demographic Characteristics

Forty-one of the study participants completed the entire training program and represented public health leaders from Hubei, Henan, Hunan, Jiangxi, and Anhui. Most respondents were male (*N *= 36, 87.80%) and over half (*N *= 34, 82.93%) had earned a bachelor's degree or master's, of which one-fifth possessed MPH degrees. Additionally, most participants (*N *= 33, 80.49%) had more than five years of experience as public health leaders. Some (*N *= 27, 65.85%) had participated in interrelated training, but the previous trainings had been conducted about 12–24 months prior to the study. The results of reliability assessment have shown that test-retest reliability and the internal consistency of questionnaires was accredited to some extent (Test-retest reliability of pre-training = 0.87, Cronbach's alpha > 0.63, respectively). The results of related analysis indicated that the construct validity of the questionnaires is of high quality (Related coefficient fluctuated between 0.37 and 0.79, *P *< 0.05) [21,22].

### Knowledge Levels

**Figure 2:**
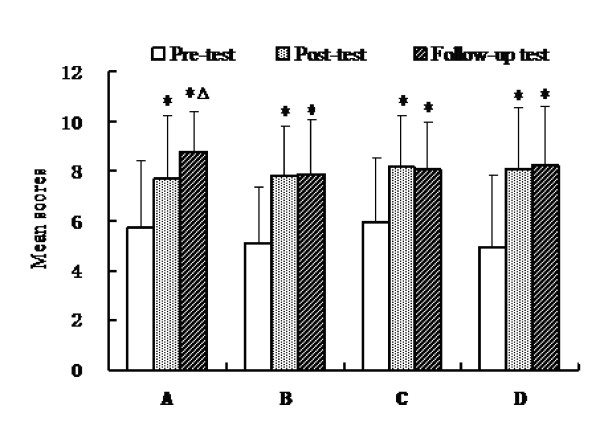
**The mean knowledge scores of participants at pre-training, post-training, and follow-up time periods (*N *= 41). **Data are shown as mean ± SD. All comparisons were performed by one way ANOVA. Abbreviations: A: Emergency analytic/assessment knowledge; B: Basic public health science knowledge; C: Basic culture knowledge; D: Program planning and management knowledge; Pre-test: The mean scores of pre-training; Post-test: The mean scores of post-training; Follow-up test: The mean scores of 12-month later. Symbols: *P < 0.05 *vs*. Pre-training; ^Δ^*P *< 0.05 *vs*. Post-training.

Results revealed that knowledge levels regarding public health emergency preparedness were relatively low before training. After the training, a significant increase was observed in the mean knowledge scores (Pre-test: 21.63 ± 6.37; Post-test: 31.59 ± 5.85; Follow-up test: 32.29 ± 5.15) (*P *< 0.01). Basic public health science and culture knowledge scores declined slightly in the follow-up test compared with that of the post-test (*P *> 0.05), but emergency analytic/assessment knowledge scores were dramatically increased in the follow-up test compared with that of the post-test (*P *< 0.05). Furthermore, overall knowledge scores were significantly increased in the follow-up test compared with the pre-test. (*P *< 0.01) (Fig. 2).

### Skills levels

Descriptive statistics on the self-assessment of skills at pre-test, post-test and follow-up test are presented in Table 2. As mentioned earlier, the responses ranged from high (5) to low (1). The results showed that participants reported a significant improvement in their skills in all fifteen competency areas examined in post-test as compared with pre-test (*P *< 0.01). The greatest improvements were reported in the leaders' ability to "use visual representations of data to identify public health problems" (an improvement in the mean score from 2.66 to 3.83, *P *< 0.01), "lead and participate in groups to identify public health problems"(2.95 to 4.07, *P *< 0.01), "cope with and lead changes in public health practice"(3.05 to 4.12, *P *< 0.01) and to "use the media and other forums to inform, educate, and empower people about health issues" (2.98 to 4.02, *P *< 0.01). Smaller improvement was detected in the leaders' ability to "match the skills and knowledge of public health workers with appropriate tasks" (3.15 to 3.68, *P *< 0.05). Twelve months later, there was a slight decline in some competency areas examined as compared with post-test (*P *> 0.05), but the mean scores had dramatically increased in all fifteen competency areas examined as compared with pre-test (*P *< 0.01).

**Table 2:** Change in self-assessment of skills among the study respondents

Self-assessment of skills	skills Level, Mean(SD)		
	
	Pre-test	Post-test	Follow-up test
Cope with and lead changes in public health practice	3.05(1.07)	4.12(0.75)*	4.07(0.82)*
Match the skills and knowledge of public health workers with appropriate tasks	3.15(0.96)	3.68(0.85)*	3.76(0.86)*
Deal with cultural and ethnic diversity in the context of access to health services	2.95(1.00)	3.85(0.82)*	3.83(0.63)*
Mobilize resources in the community needed to increase access to public health services	2.73(0.87)	3.71(0.78)*	3.76(0.80)*
Communicate clearly and effectively public health laws and regulations	2.76(0.89)	3.66(0.85)*	3.76(0.86)*
Advocate for the enforcement of laws and regulations pertaining to public health	2.88(0.95)	3.80(0.84)*	3.78(0.85)*
Understand the administrative, social, and political implications of alternative policy options	2.54(0.92)	3.49(0.71)*	3.68(0.69)*
Work with, coordinate, and/or lead community efforts to address public health problems	2.88(0.81)	3.80(0.71)*	3.76(0.70)*
Build strong and ongoing relationships with the community	2.95(0.86)	3.90(0.80)*	3.93(0.85)*
Interact, inform, and educate individuals from diverse backgrounds	2.93(0.98)	3.85(0.91)*	3.83(0.67)*
Use the media and other forums to inform, educate, and empower people about health issues	2.98(0.88)	4.02(0.72)*	3.98(0.79)*
Collaborate with colleagues and the community to manage and investigate public health problems	3.05(1.05)	3.80(0.87)*	3.76(0.77)*
Accurately and effectively communicate information to a professional and a lay audience	2.93(0.96)	3.83(0.80)*	3.85(0.76)*
Lead and participate in groups to identify public health problems	2.95(0.86)	4.07(0.72)*	3.98(0.79)*
Use visual representations of data to identify public health problems	2.66(0.94)	3.83(0.74)*	3.95(0.74)*

Note: The ordinal scale ranged from 1 to 5 (1 = "very low", 2 = "low", 3 = "Average", 4 = "high", 5 = "very high").

Abbreviations: SD: standard deviation; Pre-test: the mean scores before training; Post-test: the mean scores after training; Follow-up test: the mean scores of 12-month after training. Symbols:*P < 0.05 *vs*. Pre-training; ^Δ^*P *< 0.05 *vs*. Post-training.

### Participant satisfaction

The results of the semi-structured interview showed that participants indicated a perception of high quality for the training methods, contents, presentations, instructors' responsiveness and value, and the sessions overall. Most participants (*N *= 37, 90.24%) thought these training methods were excellent or very good, and that made the training contents could be understood clearly and easily, and 85.37% (*N *= 35) of participants were satisfied with the trainers' performance. Analysis of results showed that more than ninety-five percent of participants (*N *= 39) reported that the training model was scientific and feasible. Additionally, most participants (*N *= 40, 97.56%) were very satisfied with the venue, training logistics and services, and only one participant (*N *= 1, 2.44%) suggested logistics and services should be improved.

### Leadership response to avian influenza outbreaks

Previous evaluation of the leadership training program highlighted the participants' enhanced knowledge and changed attitude. Unexpectedly during the follow-up survey period, an outbreak of avian influenza occurred in Anhui and Hunan provinces, and two cases of human infections with avian influenza cases were confirmed, one of whom died. More than ten thousand poultries were infected and over fifty thousand poultries were killed [23,24]. Some leaders then participating in the emergency preparedness training from the Anhui and Hunan were involved firsthand in the outbreak response, which included the organization, management, operations of emergency response, etc. Although the leadership training program had no plans to evaluate the scope and dynamics of this outbreak response, the effectiveness of the leadership training was shown indirectly. The competency of the leaders, who participated in the emergency preparedness training from the Anhui and Hunan provinces, was highly praised. By personal communication, participants' colleagues and superior leaders indicated that this emergency preparedness training not only improved the capability of public health leaders to perform their task efficiently and effectively, but also brought about attitudinal changes.

## Discussion

Studies showed that continuous education and training is a process of updating knowledge, developing skills, bringing about attitudinal changes, and improving the emergency competency of participants to perform their tasks efficiently and effectively [25,26]. However, an effective and feasible training model is key to the success of an emergency training program. The leadership training program was designed strategically, which was an iterative and continually evolving process. As demonstrated by the feedback loops in the model (Fig. 1), the leadership training continuously strives to improve the system and enhance the preparedness of the public health leaders in responding to emergencies [13]. First, the needs assessments were conducted by individual and organizational analysis, and based on this information, instructional objectives, specifying what is to be achieved in the training, were developed. Second, the curriculum was developed in consultation with key stakeholders and was modified to suit the leaders' backgrounds and project needs. Third, the interactive training methods might be helpful in increasing the quality of the training and improving retention of knowledge through immediate reinforcement of learning [27-30]. Fourth, the strategic definition of projects encouraged the integrated functioning of emergency responders from public health. Through training, the results showed that the curriculum could empower public health leaders to influence state-level policy makers concerning the organization, management, and operations of emergency response. The results of follow-up also showed a significant improvement in the knowledge levels and skills of leaders who participated in the training. This suggests that it was an effective and feasible training model.

In addition, we observed an important result in that the mean scores of emergency analytic/assessment knowledge increased instead of declining during the 12-month follow-up period. This is similar to results found by Qureshis KA et al [31]. For this kind of phenomenon, we must take into consideration the experience of some public health leaders who participated in avian influenza outbreaks during the follow-up survey in 2007, which would provide practice and increase perceived relevance of the training and would likely positively affect training effectiveness. Nevertheless, the increased overall knowledge score and the positive change of skills suggested that training programs on emergency preparedness resulted in knowledge gaining and emergency capacity shifting. This is also similar to results found by Potter et al [9].

Study limitations should be noted. First, the sample frame construction was limited to the leaders that were from public health organizations/agencies, and did not include officials from other organizations/agencies. Second, evaluations were based on changes over time without the use of a horizontal comparison group. Finally, the composite variable created and used in the know-groups validity analysis may overestimate the effectiveness of training. Thus, it was not possible to fully determine which changes were due to the leadership training program and which were the result of other factors. These results, however, remained constant throughout, which provided evidence that these changes were due to the training program.

## Conclusion

The leadership training model allows for a systematic approach to assessing, designing, developing, implementing, and evaluating training that is both competency-driven and practice-based. Furthermore, the model is a closed-loop system, and the continuous monitoring and evaluation could improve the effectiveness of training. In summary, the emergency preparedness training met its aims and objective at a satisfactory level, and improved the capability of public health leaders, and developed and delivered an effective and feasible leadership training model.

## Competing interests

The author(s) declare that they have no competing interests. If accepted for publication, permissions to reproduce published material or personal communications in all forms and media are granted to BioMed Central.

## Authors' contributions

Nie SF participated in the design of the study and supervised all aspects its implementation. Wang CJ participated in the design of the study and led the writing and the statistical analysis. Wei S and Xiang H participated in the design of the study and completed the semi-structured interview. Wu J and Xu YH carried out the anonymous questionnaires and performed the statistical analysis. Liu L assisted with the study and completed the statistic analyses. All authors helped to conceptualize ideas, interpret findings, read and approved the final manuscript.

## Pre-publication history

The pre-publication history for this paper can be accessed here:


